# Case Report: From coma to genetic insights: identification of a novel pathogenic variant in Chinese neonatal CTLN1

**DOI:** 10.3389/fped.2025.1593427

**Published:** 2025-08-05

**Authors:** Lijing Deng, Yaping Liu, Kai Chen, Jianwu Qiu, Lu Chang, Junxia Xia, Yanrong Wang

**Affiliations:** ^1^ Department of Neonatology, Shenzhen Third People’s Hospital (The Second Affiliated Hospital, Southern University of Science and Technology), Shenzhen, Guangdong, China; ^2^ Department of Medical Imaging, The Fourth People's Hospital of Shenzhen (Shenzhen Samii Medical Center), Shenzhen, Guangdong, China; ^3^ Department of Neonatology, The Affiliated YueBei People’s Hospital of Shantou University Medical College, Shaoguan, Guangdong, China; ^4^ Department of Obstetrics, Shenzhen Third People’s Hospital (The Second Affiliated Hospital, Southern University of Science and Technology), Shenzhen, Guangdong, China

**Keywords:** arginine succinate synthase gene (*ASS1*), splice-site variant, minigene analysis, case report, citrullinemia type I (CTLN1)

## Abstract

Citrullinemia type I (CTLN1) is an autosomal recessive disorder caused by variants in the arginine succinate synthase gene (*ASS1*). These variants result in arginine succinate synthase deficiency, leading to a disruption of the urea cycle and hyperammonemia. To date, only a handful of CTLN1 cases have been reported in China. One neonate responded poorly 30 h after birth and progressed to coma several hours later. Family history revealed that the neonate's older brother had also died a few days after birth. Biochemical tests on admission confirmed hyperammonemia and elevated levels of citrulline and urinary orotic acid-3. Genetic analysis revealed that the parents were carriers of two heterozygous variants in *ASS1*, c.910C>T(p.Arg304Trp) and c.839-1G>A, respectively. However, the splice site variant c.839-1G>A was not present in the control databases. Minigene analysis of the c.839-1G>A resulted in the product of r.839del [p.(Gly280Valfs*15)]. In conclusion, we have identified a case of CTLN1 and diagnosed a novel pathogenic variant in the *ASS1* gene, c.839-1G>A, expanding the variant spectrum of *ASS1*. Currently, there are few reports of CTLN1 cases featuring such severe clinical manifestations and an onset at such a young age.

## 1 Introduction

Citrullinemia type I (CTLN1) is an autosomal recessive disorder characterized by hyperammonemia and neurological dysfunction, resulting from a defect in argininosuccinate synthase, a key enzyme in the urea cycle. The estimated incidence ranges from 1 in 44,300 to 200,000, as determined by neonate screening tests using mass spectrometry (1). The disorder is caused by variants in the *ASS1* gene, which spans 56 kb on chromosome 9q34.11, encodes a protein of 412 amino acids, and consists of 16 exons (1, 2), the resulting ASS protein is highly conserved throughout evolution. The study has shown that diagnosing citrullinemia through early neonatal screening and timely receiving specific treatment can avoid or reduce damage to the nervous system caused by hyperammonemia (3).

Few Chinese patients with CTLN1 have been reported, and variants in the *ASS1* gene have been identified sporadically in China (4). Here, we report a extremely rare sporadic case of CTLN1 in a Chinese neonate who developed symptoms 30 h after birth, mainly characterized by poor reaction, coma, hyperammonemia, and hypercitrullinemia. Such a young age of onset and such severe symptoms are uncommon in cases of CTLN1. Genetic sequencing revealed that the neonate had compound heterozygous variants in the *ASS1* gene, one of which was a novel, previously unreported variant site. We performed a minigene functional analysis of this new variant site.

## 2 Materials and methods

### 2.1 Subject

The subject of this research was a patient and his family members. The clinical information was collected and presented in this paper as a case report. The research was conducted in accordance with the guidelines of the World Medical Association Declaration of Helsinki (WMADH, 2008) and was approved by the Medical Ethics Committee of the Third People's Hospital of Shenzhen, China (Shenzhen Third People's Hospital Research Ethics Review No. 2022-085-02). Informed consent was obtained from the patient's parents involved in the study.

### 2.2 Next generation sequencing

Blood samples were taken from the infant and his parents, and whole-exome gene sequencing was performed using high-throughput sequencing (Shenzhen Huada Medical Laboratory). First, the DNA was fragmented and libraries were prepared. Then, the DNA in the exon and the adjacent shear region of the target gene was captured and enriched by Roche KAPA HyperExome chip, and finally, the MGISEQ-2000 sequencing platform was used for variant detection. The quality control indexes of the sequencing data were: the average sequencing depth of the target region was ≥180X, and the proportion of loci with an average depth of >20X in the target region was >95%.

### 2.3 In silico analysis

The minor allele frequency (MAF) of the variant in the population was searched in various population databases, including the 1,000 Genomes Project (1KGP, http://browser.1000genomes.org), the Exome Sequencing Project (ESP, https://evs.gs.washington.edu/EVS/), ClinVar (https://www.ncbi.nlm.nih.gov/clinvar) and the Genome Aggregation Database (gnomAD, http://gnomad.broadinstitute.org). Pathogenicity prediction was performed using the online tool Splice AI (https://spliceailookup.broadinstitute.org/).

### 2.4 Minigene analysis

We investigated the effects of the novel splice site variant in the patient using an *in vitro* splicing experiment. Primers AS13F-EcoRI (5′-ACCGGAATTCGGCTTGTCTCCTGTCAGACG-3′, forward) and AS13R-EcoRV (5′-ATTGATATCGAAGGTGCTGATACGGAGGG-3′, reverse) containing restriction sites (EcoRI/EcoRV) were designed for the variant. The target exon and flanking intronic sequences spanning the variant were amplified using the patient's maternal DNA as template. The PCR products yielded a normal and a homozygous mutant DNA fragment. We then constructed wild-type (WT) control and mutant minigene vectors by ligating the pSPL3 plasmid (BioVector NTCC, Beijing, China) with the above PCR products. Following the experimental method described by Lin et al. (5), after plasmid amplification in Escherichia coli and plasmid extraction, the sequences and correct orientations of all constructs were validated by Sanger sequencing. We used Lipofectamine 3,000 (Invitrogen) to transfect wild-type, mutant and empty vectors into HEK293t cells. After 48 h, all RNA was extracted and cDNA was generated by reverse transcription. The three groups of cDNA were amplified by PCR using the universal primers SD6 (5′-TCTGAGTCACCTGGACAACC-3′, forward) and SA2 (5′-ATCTCAGTGGTATTTGTGAGC-3′, reverse) of the pSPL3 plasmid and subjected to gel electrophoresis.

## 3 Results

### 3.1 Clinical report

A male neonate, 42 h old, was admitted to the hospital on an emergency basis because of “poor response for half a day, confusion and cyanosis for 15 min”. He was delivered by caesarean section at a gestational age of 37 weeks and 2 days due to his mother's scarred uterus, with clear amniotic fluid and no abnormalities in the placenta or umbilical cord. Apgar scores were 9 at 1 min, 10 at 5 min and 10 at 10 min. Birth weight was 2,940 g. The neonate was fed formula after birth. Half a day earlier, at 30 h of age, the neonate had a poor reaction and weak crying, but vital signs were stable, with no signs of fever, convulsions or vomiting. The family did not pay much attention. Fifteen minutes earlier, the neonate had developed cyanosis of the lips and started to moan. Pink frothy sputum and traces of milk were found in the mouth and nostrils. There was little response to stimulation. The airway was immediately opened and oxygen was administered. The neonate was then quickly transferred to the neonatal intensive care unit. His parents were not consanguineously related, and both were in good health. The neonate has an older brother who was also delivered via cesarean section seven years ago but passed away eight days after birth. The cause of death remains undetermined. He also has a sister who was delivered by cesarean section four years ago and is in good health.

On admission, the neonate's physical examination revealed a body temperature of 36.1°C, a pulse rate of 115 beats per minute, a respiratory rate of 35 breaths per minute, a blood pressure of 82/56 mmHg, and a transcutaneous oxygen saturation of 60%–70% (without supplemental oxygen). The neonate was comatose, with pale skin and cyanotic lips and extremities. The anterior fontanel was flat and soft to palpation. Bilateral pupils were round, equal in size, and approximately 3 mm in diameter. Direct and indirect pupillary reactions to light were sluggish. Breathing was shallow, and coarse rales were audible in both lungs upon auscultation. The abdomen was flat and soft, and muscle tone in was weak all limbs.

After admission, he was treated with oxygen therapy, warmth, fasting, ceftazidime drip, glucose solution infusion and electrolyte supplementation. Blood gas analysis showed the following values: pH 6.821 (reference range: 7.35–7.45), PCO2 101 mmHg (reference range: 35–45 mmHg), HCO3- 16.5 mmol/L (reference range: 21.4–27.3 mmol/L), actual base excess (reference range: −3–3 mmol/L), glucose 10. 5 mmol/L (reference range: 3.8–5.8 mmol/L) and lactate 8.3 mmol/L (reference range: 0.5–1.6 mmol/L), indicating that he was in a state of severe metabolic acidosis and respiratory failure. He was then placed on mechanical ventilation. Blood tests showed ammonia levels (VITROS Chemistry Products AMON Slides, VITROS 5,600) up to 1,157 µmol/L (reference range: 18–42 µmol/L), C-reactive protein <5.0 mg/L, calcitoninogen 23.93 ng/ml (reference range: <0.1 ng/ml), and generally normal liver and kidney function. A chest x-ray showed thickened lung markings. Colour Doppler ultrasound of the heart, brain, liver, gallbladder, spleen and bilateral kidneys and ureters showed no abnormalities. Haemodialysis was recommended due to the significant increase in blood ammonia, but his parents refused. Tandem mass spectrometry analysis of blood amino acids and acylcarnitines (LC-MS/MS, SCIEX API 3200MD) performed on day 3 after birth revealed abnormal levels of several amino acids, in particular a significant increase in citrulline levels (Table 1). The remaining amino acids, including arginine, and all acylcarnitine levels tested were normal. Subsequent urinary organic acid analysis (GC-MS, Shimadzu QP2020) on day 6 after birth revealed elevated levels of several organic acids, including 3-hydroxybutyric acid, 3-oxoproline, orotic acid and 3-hydroxybenzene lactate (Table 1). Other urinary organic acids were generally normal. An electroencephalogram (EEG) performed on day 9 showed a large number of spike waves and frequent fast and slow wave complexes, with no significant change in brain waves on stimulation.

**Table 1:** Abnormal biochemical results in blood and urine samples.

Tests	Result (μM)	Reference range (μM)	Sample source
Alanine	1,007.58	100–450	Blood
Glutamic acid	429.22	75–300	Blood
Methionine	104.67	9.5–45	Blood
Phenylalanine	145.99	25–120	Blood
Tyrosine	438.55	25–250	Blood
Leucine	266.48	60–250	Blood
Tryptophan	89.29	20–70	Blood
Citrulline	629.25	5–30	Blood
Glycine	699.20	100–500	Blood
Glutamine	111.29	1.5–85	Blood
Proline	6,265.02	450–2,700	Blood
3-Hydroxybutyric acid-2	54.3	0–9	Urine
3-Oxyproline-2	105.5	0–10	Urine
Orotic acid-3	157.5	0–2	Urine
3-Hydroxyphenyllactic acid	274.6	0–20	Urine

On the eleventh day, the neonate remained comatose with weak spontaneous respiration and was discharged due to a refusal of treatment. Unfortunately, the neonate died 36 h after discharge.

### 3.2 Next generation sequencing result

**Figure 1:**
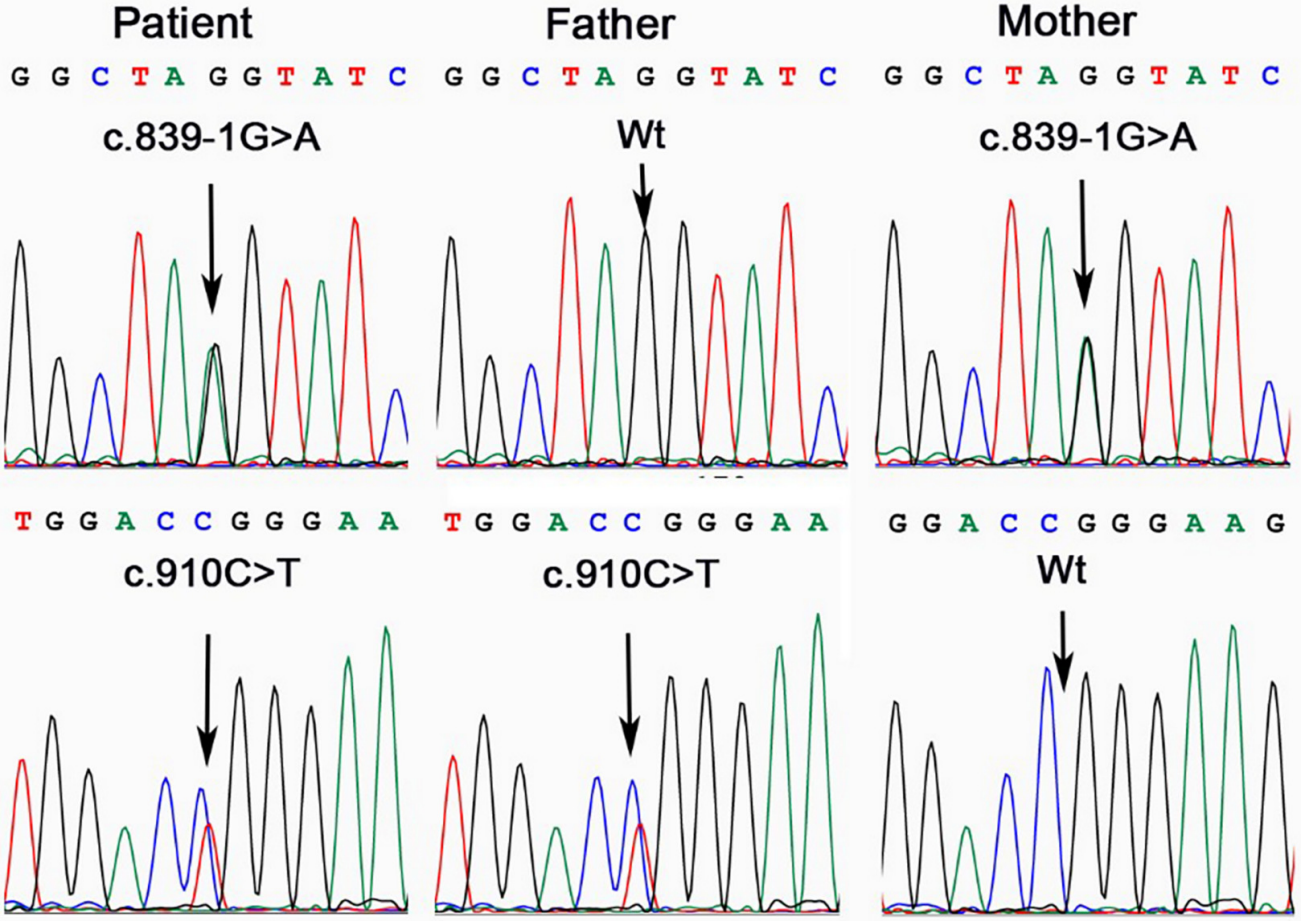
*ASS1* gene analysis in the family affected by CTLN1. The patient was a compound heterozygote of variant c.839-1G>A and c.910C>T. His father was a carrier of c.910C>T, while the mother, of c.839-1G>A.

The results revealed two heteroallelic *ASS1* variants: c.910C>T(p.Arg304Trp) and c.839-1G>A, inherited from each parent (Figure 1). The variant c.910C>T (p.Arg304Trp) has been reported (6, 7) and confirmed as a pathogenic variant according to ACMG (American College of Medical Genetics), ClinVar, and UniProt classifications, with a residual activity of less than 5% (8). In contrast, the c.839-1G>A variant has not been previously reported in the literature.

### 3.3 In silico analysis

The c.839-1G>A variant was not found in the 1,000 Genomes Project (1KGP), Exome Sequencing Project (ESP) and gnomAD databases, but was submitted once as possibly pathogenic in ClinVar (ClinVar ID: VCV002679031.1). Based on the results of the SpliceAI analysis, the variant is likely to cause a loss of the acceptor site with a significant effect (ΔScore = 0.89). This could affect the normal splicing of the *ASS1* gene, leading to abnormal transcripts, which in turn could affect the function of the protein.

### 3.4 *In vitro* splicing analytic result

**Figure 2:**
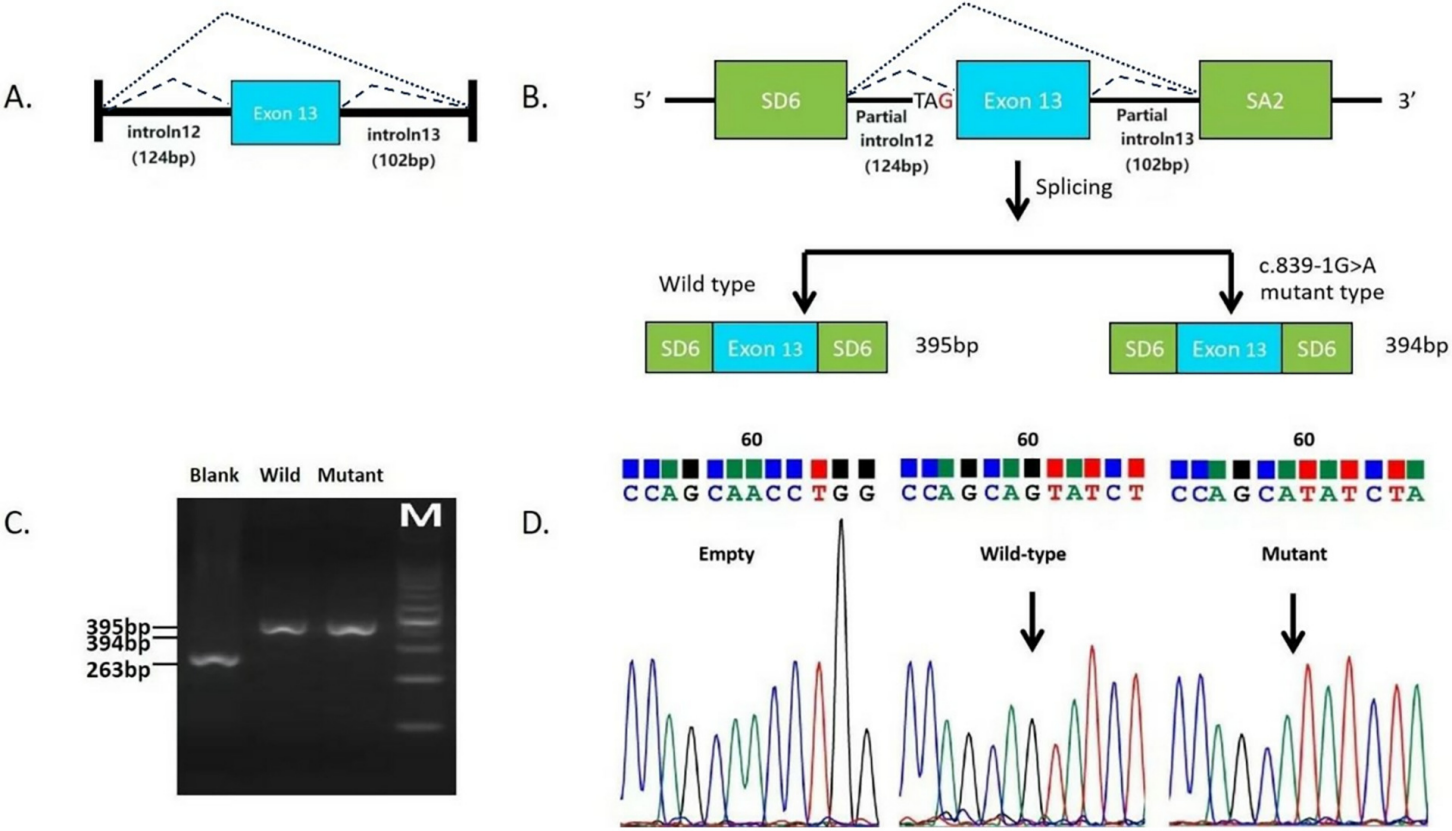
*In vitro* minigene splicing assay of variant c.839-1G>A. **(A)** Schematic representation of the DNA fragments. **(B)** Schematic representation of the mutant splicing models of c.839-1G>A. **(C)** RT-PCR products of the empty vector, wild-type vector, and mutant vectors. **(D)** Direct sequencing of the RT-PCR products of the empty control plasmid, wild-type minigene, and the mutant minigene.

A 358 bp fragment of the *ASS1* gene (Figure 2A), comprising part of intron 12 (124 bp), exon 13 and part of intron 13 (102 bp), was used to construct minigenes. These were sequenced using the Sanger sequencing method (Figure 2B). Gel electrophoresis results showed that the RT-PCR products of the empty, wild-type and mutant vectors corresponded to bands of 263 bp, 395 bp and 394 bp, respectively (Figure 2C). Sequencing results confirmed that the mutant minigene did not recognize the original 3′ splice donor site of intron 12 during transcription, and the newly generated splice donor site resulted in a deletion of one base in the RNA (Figure 2D), yielding a product of r.839del (deletion of the first G of exon 13), which caused a frameshift from position 280 and introduced a stop codon at position 295 [p.(Gly280Valfs*15)].

## 4 Discussion

CTLN1's clinical manifestations can be broadly categorized into three types: the classic type (also known as the neonatal type), the mild type (or delayed type), and the asymptomatic type (9). Patients with the classic type typically develop hyperammonemia shortly after birth and may experience symptoms such as lethargy, feeding difficulties, and vomiting. Without timely intervention, as hyperammonemia worsens and other toxic metabolites accumulate, intracranial pressure can increase, potentially leading to convulsions, coma, and even death. The mortality rate for patients with neonatal onset is very high, and many survivors suffer from neurological sequelae. The severity of these sequelae correlates with the level of blood ammonia concentration (10). In our case, the patient experienced an acute onset, characterized by poor responsiveness on the 30 h after birth, which quickly progressed to coma 12 h later. EEG revealed a large number of spike waves and frequent sharp and slow wave complexes. Laboratory tests revealed severe hyperammonemia, significantly elevated blood citrulline levels, and increased urine orotic acid, consistent with the classic clinical presentation of citrullinemia type I. This diagnosis was further confirmed by genetic sequencing. To the best of our knowledge, CTLN1 cases involving such severe clinical manifestations and an onset at such a young age are rare.

Our study identified biallelic variants in the *ASS1* gene, specifically c.910C>T (p.Arg304Trp) and a novel splice site variant, c.839-1G>A. The minigene splicing assay revealed that the variant c.839-1G>A caused the spliceosome to fail to recognize the splice donor site at the original 3′ end of intron 12, resulting in the activation of a newly generated cryptic splice donor site 1 bp upstream of the wild-type sequence. This aberrant splicing event induced a frameshift mutation starting at position 280, introducing a premature stop codon at position 295 (p.Gly280Valfs15). The results of the minigene are consistent with the clinical presentation of the child. Of course, it might be convincing if RNA could be extracted from primary cells to verify the spliced form of the c.839-1G>A variant. The minigene assay, while demonstrating a dominant 1 bp deletion event, may not fully recapitulate *in vivo* splicing complexity, such as potential exon skipping or partial retention. However, the observed frameshift mutation aligns with the severe clinical phenotype, supporting its pathogenic role.

According to the ACMG/AMP guidelines (11, 12), the novel variant c.839-1G>A is a splice-site variant at the −1 position, which represents a loss-of-function variant. The SpliceAI score is extremely high (0.89), constituting strong evidence of pathogenicity, aligning with evidence classified as PVS1_Strong. Minigene analysis confirmed that the variant causes premature termination of the peptide chain and affects the function of the corresponding protein, this experimental evidence fulfills the criteria for PVS1_Strength (RNA). This new variant locus has an allele frequency of 0 in the ESP database, the 1,000 Genomes Project and the ExAC database, fulfilling the PM2_Supporting criterion. CTLN1 is an autosomal recessive disease. A compound heterozygous variant was detected in the proband and the pathogenicity of one of the variants, c.910C>T (p.Arg304Trp), contributing to the compound heterozygosity, was established, fulfilling criterion PM3. The infant presented with hyperammonemia, hypercitrullinemia and severe neurological problems, including coma and abnormal EEG, clinically supporting the diagnosis of CTLN1, fulfilling criterion PP4. Considering all these factors, the novel variant identified in this study is classified as a pathogenic variant [PVS1_Strength (RNA) + PM2_Supporting + PM3 + PP4].

Notably, while a variant at c.839-2A>T(ClinVar ID: VCV001068237.7) in ASS1 is classified as “Likely Pathogenic” in ClinVar, its pathogenicity assertion lacks experimental evidence of splicing disruption. According to the recommendations of Walker et al. (12), variants in the core splice site region (such as the −1 position GT dinucleotide) are assumed to have strong pathogenicity (PVS1_Strong) due to direct disruption of spliceosome recognition, whereas variants at non-core positions like −2 require experimental evidence to upgrade their pathogenicity score. In this study, the c.839-1G>A variant was confirmed to cause abnormal splicing through minigene assays (Figures 2C,D), activating a cryptic donor site 1 bp upstream and generating a novel NAG acceptor motif (r.839del) and a truncated protein (p.Gly280Valfs*15). This meets the classification criteria of “PVS1_Strength (RNA) supported by experimental evidence” proposed by Walker et al. (12), demonstrating significantly stronger pathogenicity than the c.839-2A>T variant lacking functional evidence. This distinction highlights the importance of functional validation in differentiating splice-site variants, particularly when relying solely on positional annotations in database entries like ClinVar.

The molecular genetic diagnosis of this infant was completed after the death of the neonate, and the molecular diagnosis did not guide the clinical treatment and care during the infant's illness. This was the second neonate with unexplained cause death in the unfortunate family. Exome sequencing can provide irreplaceable molecular evidence for genetic counselling (e.g., clarifying the risk of mutation carrying in the family lineage, and guiding reproductive decision-making) through the identification of the causative genotypes. It can provide irreplaceable molecular evidence for future precision medicine (e.g., potential gene therapy enrollment). In addition, the infant's family was deeply puzzled by the infant's symptoms, and even questioned the prenatal examination and obstetrician, which was dispelled after the cause of the disease was clarified through molecular diagnosis.

Clinically, neonatal intrahepatic cholestasis caused by Citrin deficiency (NICCD) can also lead to elevated citrulline levels. However, NICCD is generally not life-threatening, it is primarily characterized by intrahepatic cholestatic jaundice as the main clinical manifestation, including low birth weight, growth restriction, intrahepatic cholestasis, diffuse fatty liver, hepatomegaly, parenchymal cellular infiltration associated with hepatic fibrosis, hypoglycemia, hypoproteinemia, hyperammonemia (rarely severe), coagulopathy, and liver dysfunction. The clinical manifestations of NICCD improve or even resolve spontaneously by the age of 12–24 months in most patients (13). Notably, neurological impairment rarely occurs in the neonatal period of NICCD. Ultimately, genetic diagnosis can be used to identify NICCD and CTLN1.

In conclusion, we report a case of a Chinese neonate with CTLN1 who developed symptoms 30 h after birth, with rapid disease progression and a poor prognosis. This is one of the younger age of onset patients with severe clinical manifestations of CTLN1 reported worldwide to date. The novel splice site variant c.839-1G>A identified in this study was shown to be pathogenic by *in vitro* functional studies. This study expands the spectrum of *ASS1* variants and provides a reliable molecular marker for definitive diagnosis and genetic counselling of CTLN1 in affected families.

## Data Availability

The original contributions presented in the study are included in the article. Further inquiries can be directed to the corresponding author.
